# Metagenome analyses identify human endogenous retrovirus–K113 (HML-2) subtype in glioblastoma

**DOI:** 10.1172/JCI173959

**Published:** 2023-12-15

**Authors:** Amanda Macamo, Jan Beckervordersandforth, Axel zur Hausen

**Affiliations:** Department of Pathology, GROW - School for Oncology and Reproduction, Maastricht University Medical Centre, Maastricht, Netherlands.

**Keywords:** Oncology, Virology, Brain cancer

## To the Editor:

We read with great interest the article by Shah et al., demonstrating human endogenous retrovirus K (HERV-K; HML-2) overexpression in glioblastoma multiforme (GBM) and its role in maintaining stemness and tumorigenesis (1).

**Figure 1:**
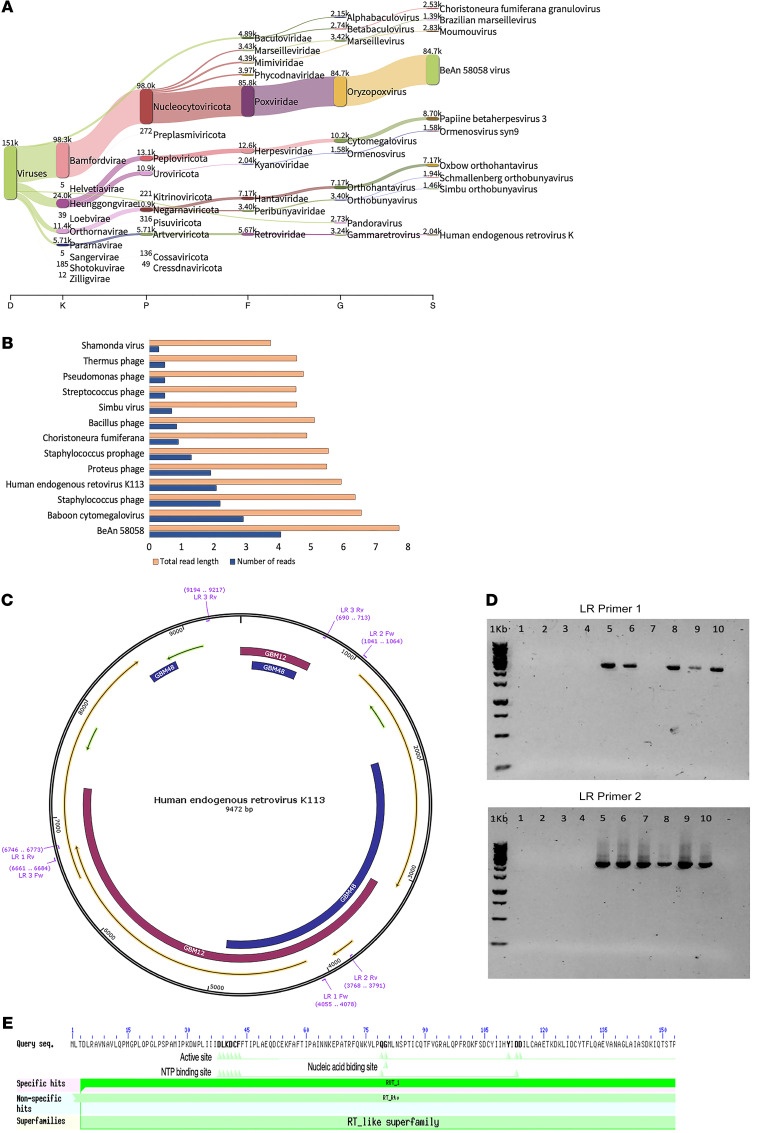
Identification of HERV-K113 in GBM. (**A**) Centrifuge metagenomics classification analysis. D, dogma; K, kingdom; P, phylum; F, family; G, genus; S, species. (**B**) BLAST summary. (**C**) Map of locations of HERV-K113 LR primers. (**D**) Gel electrophoresis. (**E**) Conserved domain. The bold letters in the sequence, represent the part of the query sequence that is identical (hit) to the subject (RVT_1).

This article coincided with our findings from metagenomic analyses on the potential involvement of viruses in GBM etiology. Using and comparing two different bioinformatic approaches, we discovered HERV-K113 in the GBM tissues tested and validated these combined results by PCR and RT-PCR. In analyzing GBM-related samples (15 samples) downloaded from three Sequence Read Archive (15 samples) studies with the centrifuge metagenomics classification tool (2), many reads aligned to HERV-K (Figure 1A, the flow of reads from the left side of the graph through different viral taxonomic units eventually identifies a viral species on the right). Furthermore, compared with our analysis of normal brain samples (3 samples), HERV-K113 viral abundance was only detected in GBM samples. The results of this analysis were compared to our long-read sequences of GBM samples (2 samples), and we analyzed the subsequent metagenomic classification using NCBI BLAST (Figure 1B, BLAST summary of a GBM sample; the graph displays the total read lengths and number of reads for different viruses on the *y* axis, while the *x* axis shows the log scale for these variables). To confirm the outcomes of the metagenomic analyses and to evaluate which region of the HERV-K113 might be of importance, PCR primers were designed to target specific areas of HERV-K113, as identified in the alignment results of the BLAST analysis (Figure 1C).

RT-PCR was performed on the isolated RNA from these samples using long range 1 and 2 (LR1 and LR2) primers, revealing 50% and 60% positivity, respectively. (Figure 1D, results of RT-PCR using LR1 [expected product size, 2,719 bp] and using LR2 primer [expected product size, 2,750 bp]). Sanger sequencing of all PCR products confirmed HERV-K113. PCR products of LR1 revealed a sequence identity of 97% to HERV-K113 — the query sequence clustered with an integrated HERV-K (HML-2) sequence (HML-2_19p12b). Using LR1 primers, the HERV-K113 polyprotein was identified as the longest ORF on the first frame of the positive strand, with which it shared 93.5% sequence identity. Our data suggest that the HERV-K113 sequences are integrated into the GBM genomes and transcribed. Specific hits for the conserved domain of the reverse transcriptase (RVT_1) protein were identified with ORF predictions by long-range PCR products (Figure 1E).

## Version 1 12/15/2023

Electronic publication
